# Research Note: Sex difference in changes in heterophil to lymphocyte ratios in response to acute exposure of both corticosterone and cortisol in the Pekin duck

**DOI:** 10.1016/j.psj.2022.101914

**Published:** 2022-04-09

**Authors:** V. Tetel, S. Tonissen, G.S. Fraley

**Affiliations:** Department of Animal Sciences, Purdue University, West Lafayette, IN 47907, USA

**Keywords:** welfare, wellbeing, hypothalamic-pituitary-adrenal axis, immune response

## Abstract

Poultry scientists have utilized both direct and indirect measures of stress hormones for monitoring the state of avian welfare. For decades, it has been assumed that the mammalian and avian hypothalamic pituitary adrenal (**HPA**) function similarly to one another. However, there are considerable differences between the 2. Further, it has been assumed that the predominate glucocorticoid (**GC**) in birds was corticosterone, but recent studies have suggested that both corticosterone and cortisol are secreted. GC release is associated with an increase in blood heterophils due to increased migration from the lymph nodes and a decrease in lymphocytes due to marginalization. Both actions account for an increase in heterophil to lymphocyte ratios (**HLR**). The goal of this project was to determine the effect of each GC on HLR over time. To achieve this, we intramuscularly injected 2.0 mg/kg of corticosterone or cortisol, a lower dose cortisol treatment (0.5 mg/kg), or safflower oil as vehicle control. Blood was collected prior to intramuscular (**IM**) injections and blood collected 3 more times at every hour. Blood smears were also collected to assess HLR at the same four time points. HLR assays were completed by avian pathologists from an independent lab who were unaware of the treatments. Data were analyzed by 3-way repeated measures ANOVA with a *P* < 0.05 considered significant. We found significant sex (*P* < 0.001) x treatment (*P* < 0.001) x time (*P* < 0.001) effects with significant interactions (*P* = 0.0055). In hens, both GC resulted in significant increase in HLR at 1 h after injection compared to controls. In drakes, however, both GC showed a significant increase in HLR but not until 2 h after injection. The low dose cortisol had no significant effect on HLR in either sex. These data suggest that sex differences need to be considered when assessing duck welfare, and that cortisol may play a role in the HPA axis in ducks.

## INTRODUCTION

Many conflicting reports regarding the stress response in birds have been published over the decades, and there has been a large focus on assessing welfare in poultry species. Emphasis has been placed on establishing physiological variables to assess a bird's welfare status, including corticosterone and the heterophil to lymphocyte ratio. Studies that involve transportation and shackling have seen varying results in glucocorticoid (**GC**) levels (Scanes et al., 2020). A study done by Kannan et al. (1997) showed that transportation had no effect on circulating GC levels, likewise with Zhang et al. (2009) where the results showed that transport stress did not affect the HLR of the broilers in the study. In contrast, the effects of transportation stress in a study by Cheng and Jefferson (2008) revealed that certain chicken strains had higher levels of plasma corticosterone and larger adrenal glands. A study done by Scanes et al. (2020) showed that HLR actually declined with shackling birds and that the HLR response varied depending on what time of day it was, and what was even more surprising was that shackling of broilers did not increase GC concentrations or HLR. Historically, avian biologists focused on both corticosterone and cortisol as indicators of stress, but in poultry science the emphasis on cortisol has been lost. However, recent evidence suggests that cortisol is an important indicator of welfare status in birds (Ralph and Tilbrook, 2016) and we need to reconsider examining both corticosterone and cortisol. The purpose of this study was to determine the relationship of the effect of an acute injection of corticosterone or cortisol to elicit a change in the HLR.

## MATERIALS AND METHODS

### Animals

Adult breeder Pekin ducks were obtained from Maple Leaf Farms, Inc. (Leesburg, IN). They were between 35 and 40 weeks of age, with hens and drakes weighing 4 to 4.5 kg, respectively. They were housed in a barn at Purdue Animal Sciences Research and Education Center (**ASREC**). All procedures were approved by the Purdue University Institutional Animal Care and Use Committee (**PACUC**).

### Experimental Design

Adult (∼40 weeks of age) Pekin ducks were obtained from a local producer and housed in floor pens by sex at the research farm at Purdue University. Each pen consisted of a litter floor and raised plastic flooring over a pit and under the water nipple lines. Litter constituted approximately two-third of the surface area of each pen. Ducks were housed at a density (∼0.348 m^2^/duck) comparable to industry standards and with 3 ducks per water nipple. Pens for the hens also contained a bank of 3 nest boxes. Feed was a typical breeder diet and was provided for 8 hours per d, again per industry standards.

Ten ducks (N = 10/sex/treatment) were assigned to each treatment for this study: control, corticosterone, cortisol, or low-dose cortisol injections. They were injected intramuscularly (**IM**) with 2.0 mg/kg of each GC in order to assess equimolar doses of each GC. In addition, a low dose of cortisol, 0.5 mg/kg, was included to more appropriately represent the endogenous relationship of cortisol to corticosterone levels. Safflower oil was used as the control. The doses were chosen as those shown to elicit responses previously in ducks (Fowles et al., 1993) and that we have shown that circulating endogenous cortisol is typically 25% levels compared to corticosterone in ducks (Tetel et al., 2022a,b). Steroids were dissolved in safflower oil for injections. Safflower oil was used due to the undetectable levels of genistein, a plant estrogen; important because any estrogenic activity could affect the glucocorticoid responses. Blood was collected from the tibial vein using a prick with a 25 Ga needle, and a small amount of blood collected in a heparanized capillary tube to produce the blood smear. Blood smears were collected from each duck prior to injections then again at 1-, 2-, and 3-h post injection for a total of 4 blood samples per duck. Blood smears were analyzed for Complete Blood Count (**CBC**) differentials by an independent lab who were unaware of the study treatment groups. All procedures were approved by the Purdue Animal Care and Use Committee.

### Statistical Analyses

Data were analyzed by 3-way (sex x treatment x time) repeated measures ANOVA, with *P* values < 0.05 considered significant. All date were analyzed using MacJMP (SAS, JMP Pro v15).

## RESULTS AND DISCUSSION

Significant (*P* = 0.00098) increases in HLR were observed with all treatments, with a significant sex x treatment x time interactions for both GC. Hens showed significant increases in HLR at 1 h after injection of all 3 experimental treatments compared to the controls. Drakes showed significant increases in HLR but not until 2 h after injection but not in the low dose cortisol treatment.

Previous results from our lab have shown a sex difference in both corticosterone and cortisol release following shipping stress and in response to treatment with an artificial adrenocorticotropin releasing hormone (**ACTH**; cosyntropin; Tetel et al., 2022a,b). Glucocorticoids increase HLR by stimulating lymph nodes to release heterophils which elicits an apparent increase in circulating numbers while simultaneously increasing marginalization of lymphocytes which leads to an apparent decrease in numbers. The idea that cortisol may play a role in the stress response in birds is not new. Another study showed that cortisol appears to be the primary glucocorticoid to bind to bursal tissue, and it binds with high affinity to a neural membrane receptor (Schmidt et al., 2010). A recent study also demonstrated that cortisol is present in egg albumen whereas corticosterone is not (Caulfield and Padula, 2020).

**Figure 1:**
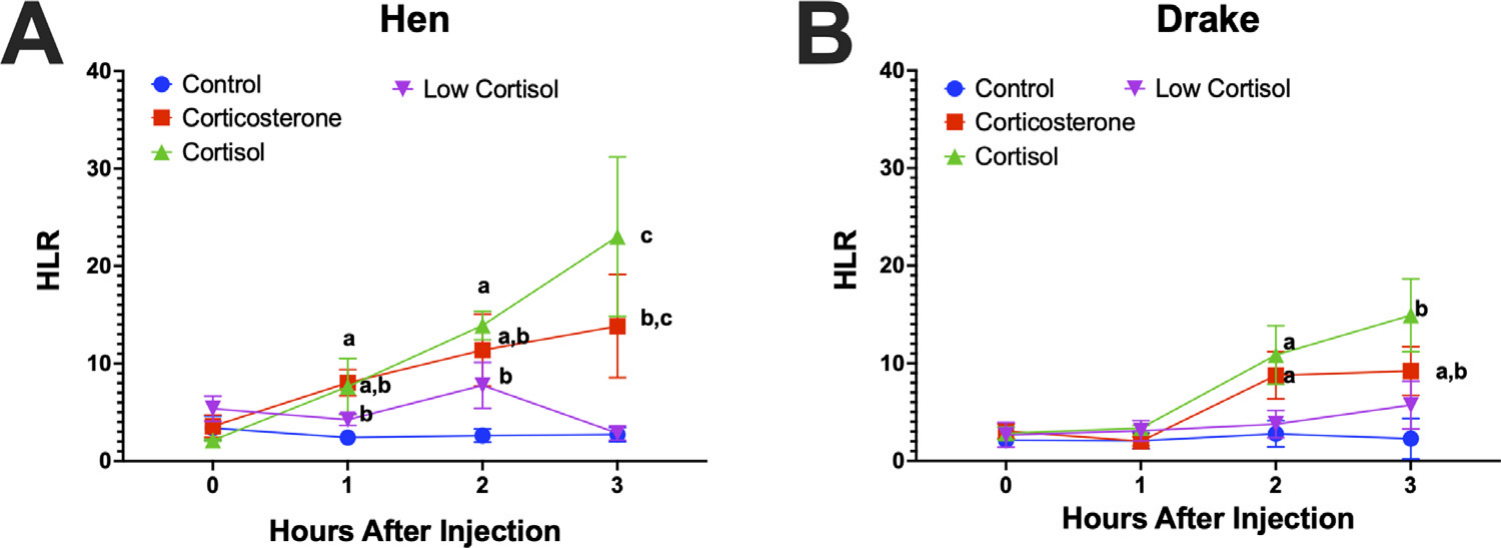
Heterophil to lymphocyte (HLR) response to acute glucocorticoid injections. Sexes are separated in two panels for clarity. (A) Hens showed significant increases in HLR at 1 hour after injection of all 3 experimental treatments compared to the controls. (B) Drakes showed significant increases in HLR but not until 2 h after injection. Letters indicate statistically different levels as determined by the ad hoc statistical tests.

Other studies have similarly shown that females tend to have higher levels of glucocorticoids than do males, particularly in mammals. Our previous studies also showed a significant sex difference in the secretion pattern of both glucocorticoids (Tetel et al., 2022a,b). The general or poor lack of effect of the low dose cortisol may suggest that corticosterone is the primary glucocorticoid to effect HLR, however, more investigations are required to confirm that notion. Combined, our data suggests first, that cortisol has biological activity in the duck, and second, that sex differences in glucocorticoid release and subsequent changes in HLR need to be considered when assessing welfare of birds relative to the onset of a stimulus. The timing of blood draws to measure glucocorticoid levels or HLR need to occur with specific attention to the timing of the birds’ perceived onset of the stressor and the sex of the bird Figure 1.

## Disclosures

The authors declare no conflicts of interest.
